# The relationship between insulin and glucagon concentrations in non‐diabetic humans

**DOI:** 10.14814/phy2.15380

**Published:** 2022-07-13

**Authors:** Marcello C. Laurenti, Praveer Arora, Chiara Dalla Man, James C. Andrews, Robert A. Rizza, Aleksey Matveyenko, Kent R. Bailey, Claudio Cobelli, Adrian Vella

**Affiliations:** ^1^ Division of Endocrinology, Diabetes & MetabolismEndocrine Research Unit, Mayo Clinic, College of Medicine and Science Rochester Minnesota USA; ^2^ Biomedical Engineering and Physiology Graduate Program, Mayo Clinic Graduate School of Biomedical Sciences Rochester Minnesota USA; ^3^ Department of Information Engineering University of Padova Padova Italy; ^4^ Vascular and Interventional Radiology, Mayo Clinic, College of Medicine and Science Rochester Minnesota USA; ^5^ Division of Biomedical Statistics and Informatics, Mayo Clinic, College of Medicine and Science Rochester Minnesota USA; ^6^ Department of Woman and Child's Health University of Padova Padova Italy

**Keywords:** cross‐approximate entropy, glucagon, insulin, prediabetes

## Abstract

Abnormal postprandial suppression of glucagon in Type 2 diabetes (T2DM) has been attributed to impaired insulin secretion. Prior work suggests that insulin and glucagon show an inverse coordinated relationship. However, dysregulation of α‐cell function in prediabetes occurs early and independently of changes in β‐cells, which suggests insulin having a less significant role on glucagon control. We therefore, sought to examine whether hepatic vein hormone concentrations provide evidence to further support the modulation of glucagon secretion by insulin. As part of a series of experiments to measure the effect of diabetes‐associated genetic variation in *TCF7L2* on islet cell function, hepatic vein insulin and glucagon concentrations were measured at 2‐minute intervals during fasting and a hyperglycemic clamp. The experiment was performed on 29 nondiabetic subjects (age = 46 ± 2 years, BMI 28 ± 1 Kg/m^2^) and enabled *post‐hoc* analysis, using Cross‐Correlation and Cross‐Approximate Entropy (Cross‐ApEn) to evaluate the interaction of insulin and glucose. Mean insulin concentrations rose from fasting (33 ± 4 vs. 146 ± 12 pmol/L, *p* < 0.01) while glucagon was suppressed (96 ± 8 vs. 62 ± 5 ng/L, *p* < 0.01) during the clamp. Cross‐ApEn was used to measure pattern reproducibility in the two hormones using glucagon as control mechanism (0.78 ± 0.03 vs. 0.76 ± 0.03, fasting vs. hyperglycemia) and using insulin as a control mechanism (0.78 ± 0.02 vs. 0.76 ± 0.03, fasting vs. hyperglycemia). Values did not differ between the two scenarios. Cross‐correlation analysis demonstrated a small in‐phase coordination between insulin and glucagon concentrations during fasting, which inverted during hyperglycemia. This data suggests that the interaction between the two hormones is not driven by either. On a minute‐to‐minute basis, direct control and secretion of glucagon is not mediated (or restrained) by insulin.

## Graphical abstract

This manuscript suggests that insulin pulses do not necessarily regulate glucagon secretion in humans.

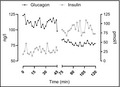

## 1 INTRODUCTION

Postprandial hyperglycemia arises from delayed and deficient insulin secretion as well as impaired suppression (or a paradoxical rise) of glucagon concentrations. The net result is a decrease in the post‐prandial suppression of endogenous glucose production (Adams et al., 2021; Shah et al., 2000). Insulin has been proposed as an important regulator of glucagon secretion but the mechanism by which this occurs remains obscure. A series of experiments, examining the interaction between these hormones, demonstrated a reciprocal relationship between glucagon and insulin concentrations (Meier et al., 2006; Menge et al., 2011). In both experiments, Cross‐ApEn was used to measure the interaction of the two hormones. It is a variation of Approximate Entropy (ApEn), a measurement of regularity and orderliness frequently applied to physiological signals (Pincus, 1991, 2001). In this case, when two separate signals are evaluated simultaneously, Cross‐ApEn measures the statistical dissimilarities between the two linked concentration time series. “Forward” cross‐ApEn where serial insulin concentrations were used as the template to assess pattern reproducibility in glucagon concentrations and “reverse” cross‐ApEn where the effect of glucagon concentrations on the pattern of insulin concentrations, were compared. Although forward cross‐ApEn was significantly higher than reverse cross‐ApEn, the experimental results were taken to imply that insulin regulates glucagon secretion. The relationship was abrogated by β‐cell destruction using alloxan in pigs (Meier et al., 2006) and was also absent in people with T2DM (Menge et al., 2011).

Subsequent to those observations, evidence has accumulated in humans that α‐cell dysregulation can occur in the presence of impaired insulin action (Faerch et al., 2016; Ferrannini et al., 2007; Sharma et al., 2018) but independently of defects in postprandial insulin secretion (Adams et al., 2020; Shah et al., 2016). This has challenged the contention that α‐cell secretion is regulated and restrained by the β‐cell under physiologic conditions (Walker et al., 2011). Indeed, given the stimulatory effect of glucagon on insulin secretion, some authors have suggested that α‐cell function is necessary for optimal β‐cell function (Finan et al., 2020).

The measurement of islet hormone pulsatility in humans is challenging, in part because they are secreted into the portal vein in a pulsatile manner. Their concentrations are also attenuated by hepatic extraction and systemic hormone clearance. One solution to these issues—measuring their concentrations in the hepatic vein, before the effect of systemic clearance—limits the ability to conduct adequately powered studies across different disease states. The other solution—measurement of pulses in the portal vein, prior to hepatic extraction—is even more invasive and poses unacceptable risks in humans (Laurenti, Matveyenko, et al., 2021). This has limited further study of the relationship between insulin and glucagon pulsatility.

Insulin and glucagon are secreted into the portal vein and undergo some degree of hepatic extraction prior to appearing in the hepatic vein. A greater proportion of insulin is extracted by the liver and the magnitude of extraction is time‐variant and dependent on the amplitude and frequency of insulin pulses (Meier et al., 2005). Unlike insulin, the impact of liver extraction on glucagon is less problematic for pulsatility studies, because hepatic clearance of glucagon is smaller and relatively fixed (Ishida et al., 1983). More importantly, it is not altered by the glycemic state or glucagon secretion (Herold & Jaspan, 1988). As part of a series of experiments examining the contribution of genetic variation in *TCF7L2* to the pathogenesis of T2DM, we previously examined insulin pulse characteristics in non‐diabetic subjects (Laurenti et al., 2020). To do so, we demonstrated that hepatic vein concentrations of insulin mirror the pulsatile characteristics of insulin secretion estimated by deconvolution from peripheral C‐peptide concentrations (Laurenti et al., 2019; Varghese et al., 2018). This is important because it validates prior work using hepatic vein insulin concentrations to measure insulin pulse characteristics (Porksen et al., 2002).

Therefore, in the current experiment, simultaneous sampling of glucagon and insulin from the hepatic vein, provides a unique opportunity to examine the relationship between insulin and glucagon during fasting and hyperglycemic conditions.

## 2 METHODS

### 2.1 Screening

After approval by the Mayo Clinic Institutional Review Board, data from 29 non‐diabetic subjects who had provided informed, written consent prior to participation in a published experiment (Laurenti et al., 2020) were utilized. At the time of study, they had no active illness or diabetes and were not taking medications that alter glucose metabolism.

### 2.2 Experimental design

The complete experimental design has been reported previously (Laurenti et al., 2019). Briefly, subjects were admitted to the Clinical Research and Translation Unit (CRTU) at 17:00 the evening prior to study. The following morning (at approximately 06:30), an 18 g cannula was inserted retrogradely into a dorsal hand vein. This was then placed in a heated Plexiglas box (55°C) to allow sampling of arterialized venous blood. Subjects were then moved to the radiology suite where a hepatic vein catheter was placed via the femoral vein under fluoroscopic guidance. Following their return, at 08:00 (0 min) blood was sampled every 2 min from the hepatic vein over 45 min (Fasting Phase). At 08:46 (46 min) glucose infusion commenced, and the infusion rate was adjusted to rapidly achieve and maintain peripheral glucose concentrations of ~9 mmol/L. Following this 30‐min interval (09:15), blood was again sampled every 2 min during the Hyperglycemic Phase for an additional 45 minutes. Glucose concentrations were measured at 5‐min intervals.

### 2.3 Analytic techniques

Plasma samples were placed on ice, centrifuged at 4°C, separated, and stored at −20°C until assayed. Glucose concentrations were measured from dorsal vein samples using a glucose oxidase method (Yellow Springs Instruments, Yellow Springs, OH). Plasma insulin was measured from hepatic vein samples using a chemiluminescence assay (Access Assay; Beckman, Chaska, MN). Plasma glucagon was measured from hepatic vein samples by Radioimmunoassay (Linco Research, St. Louis, MO).

### 2.4 Calculations and statistical analysis

Cross‐Correlation analysis was used to evaluate their interrelationship in the time window of the experiment. In each subject, the “xcross” function in MATLAB was used to calculate the maximum linear correlation coefficient between insulin and time‐shifted glucagon concentrations at increasing time lag between the paired values. The function was set to limit the maximum lag allowed to one‐fourth of the time window to avoid correlations measured on the overlapping of too small portions of the time series. A positive value of Cross‐Correlation indicates that oscillations of the two hormones are *in‐phase*, where a rise in one hormone concentration implies a significant rise in the other. Negative values of Cross‐Correlation imply that the two hormones coordinate in an *anti‐phase* fashion, where a rise in the concentration of one a hormone produces suppression of the other.

Cross‐Approximate Entropy (Cross‐ApEn) was used to study the degree of regularity and coupling between glucagon and insulin concentrations. This index measures spatial and temporal independence; a lower number of pattern matches implies a higher value of Cross‐ApEn and of asynchrony (Martinez‐Zarzuela et al., 2013). Cross‐ApEn is measured by comparing the pattern of one hormone time series (the template) with the values of the other (the follower). Therefore, it is measured twice, with insulin used as the template to assess pattern reproducibility for glucagon (implying that the insulin time series would predict, and therefore control, glucagon) and vice versa. The index is not defined for every sample match and two different corrections have been proposed (Delgado‐Bonal & Marshak, 2019). We report our results using a bias‐zero correction. However, we achieved similar conclusions when a bias‐max correction was tested (data not shown).

Data and results are presented as mean ± SEM. Time‐series analysis was performed in MATLAB 2020a (MathWorks, Natick, MA, USA). Comparison among parameters was performed using an unpaired, two‐tailed Student *t*‐test for normally distributed variables; when samples were not normally distributed, a two‐tailed Wilcoxon test was used. A *p*‐value <0.05 was considered statistically significant.

## 3 RESULTS

### 3.1 Insulin and glucagon concentrations during fasting and hyperglycemia (Figure 1)

**FIGURE 1:**
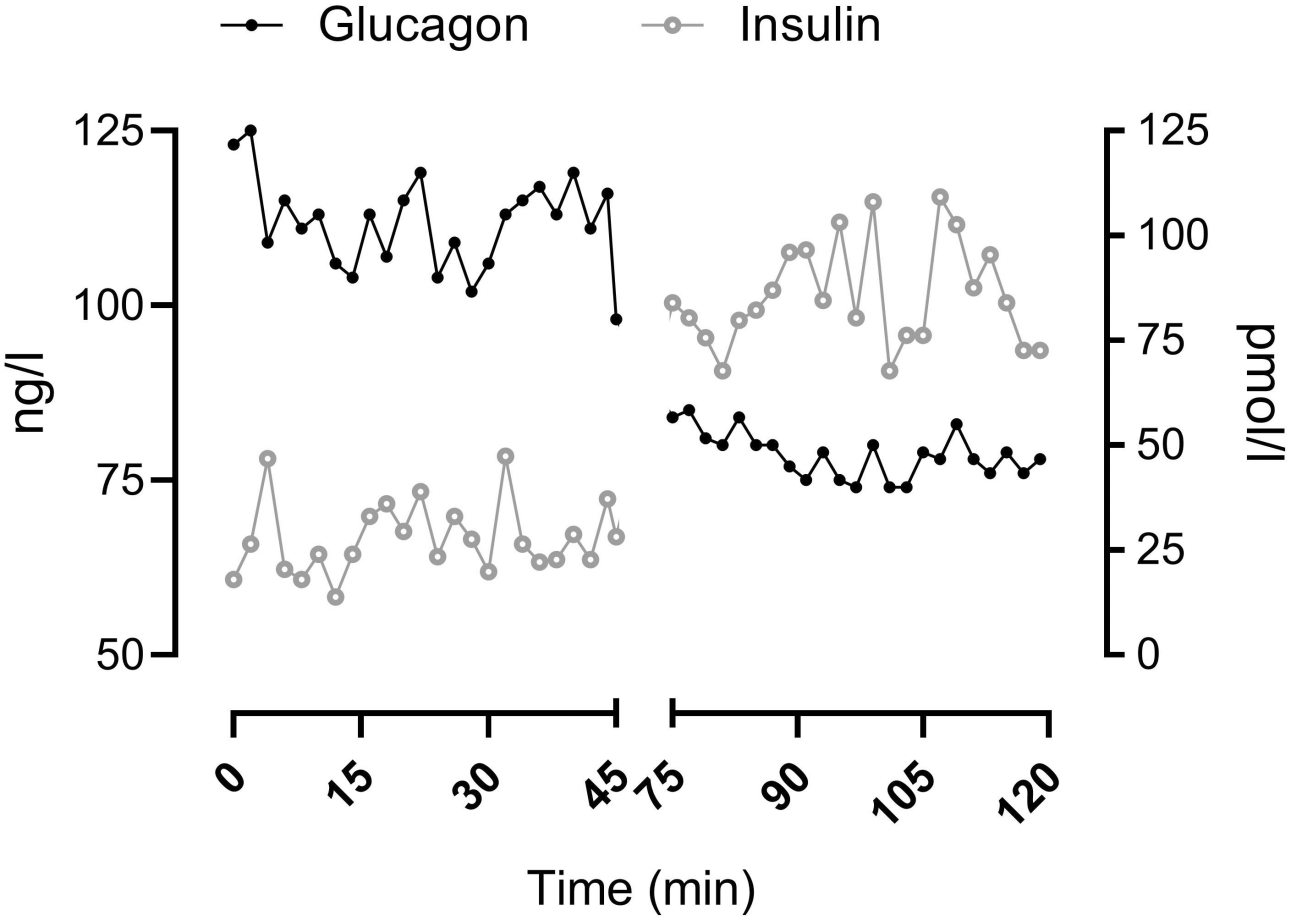
Glucose concentrations during fasting and hyperglycemia in a representative subject (Panel a). Glucagon (left *Y*‐axis) and insulin (right *Y*‐axis) concentrations of the same subject, measured in the hepatic vein during the fasting and hyperglycemic phase of the experiment (Panel b).

A total of 29 nondiabetic subjects (age = 46 ± 2, BMI 28 ± 1 Kg/m^2^) were studied. Participants had fasting glucose concentrations of 4.8 ± 0.1 mmol/L, fasting insulin concentrations averaged 33 ± 4 pmol/L, and glucagon concentrations of 96 ± 8 ng/L. During the hyperglycemic clamp, glucose rose to 8.8 ± 0.2 mmol/L, insulin rose to 146 ± 12 pmol/L while glucagon suppressed to 62 ± 5 ng/L. Data for a representative subject are shown in Figure 1, while average data are visible in (Laurenti et al., 2019).

### 3.2 Cross‐ApEn and Cross‐Correlation analysis of the relationship of insulin and glucagon during fasting and hyperglycemia (Figure 2)

**FIGURE 2:**
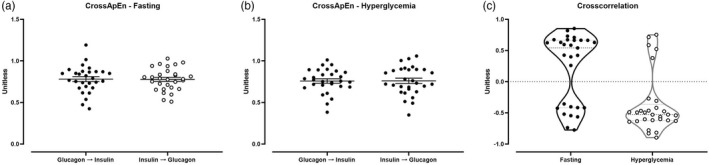
Synchronicity and correlation analysis of glucagon and insulin concentrations during fasting and hyperglycemia. Panel a: Cross‐approximate entropy statistic, calculated during fasting, using glucagon as a template to assess pattern reproducibility in insulin concentrations (glucagon → insulin) and vice‐versa (insulin → glucagon). Panel b: Cross‐approximate entropy statistic, calculated during the hyperglycemic clamp, using glucagon as a template to assess pattern reproducibility in insulin concentrations (glucagon → insulin) and vice‐versa (insulin → glucagon). Panel c: Cross‐correlation coefficients between the hormone time series, evaluated using a lag‐shifted version of glucagon over insulin concentrations.

During fasting (Panel a), there was no difference in the level of hormone interaction, as measured by Cross‐ApEn. Similar values of the index were observed when Cross‐ApEn was calculated using glucagon as the template to assess pattern reproducibility in insulin concentrations (0.78 ± 0.03) compared to when insulin was used as the template for pattern reproducibility in glucagon concentrations (0.78 ± 0.02). When performing the analysis for hyperglycemia similar results were produced for both cases (0.76 ± 0.03 vs. 0.76 ± 0.03, using glucagon and insulin respectively as templates—Panel b).

A Cross‐Correlation analysis confirmed between‐hormone synchronicity. However, the result distribution is bimodal (Panel c). During fasting, most subjects showed a positive correlation between hepatic vein glucagon and insulin concentrations (0.61 ± 0.03), with a few subjects showing an inverse relationship (−0.52 ± 0.05). This distribution inverted during hyperglycemia: the relationship was anti‐phasic (−0.57 ± 0.05) in the majority and in phase for the remainder (0.59 ± 0.07). Of note, the average time lag in the two scenarios was about half a minute, with a range of [−12, +12] minutes. This explorative approach, therefore, allows that glucagon could lead insulin in some subject and follow insulin in others. When a more conservative approach was used, assuming that glucagon was limited to only lead (negative time lag) or only follow insulin (positive time lag) in all the groups, similar correlations values and trends were observed (data not shown).

### 3.3 Effect of glucose concentration on the measurement of interplay and synchronicity between insulin and glucagon (Figure 3)

**FIGURE 3:**
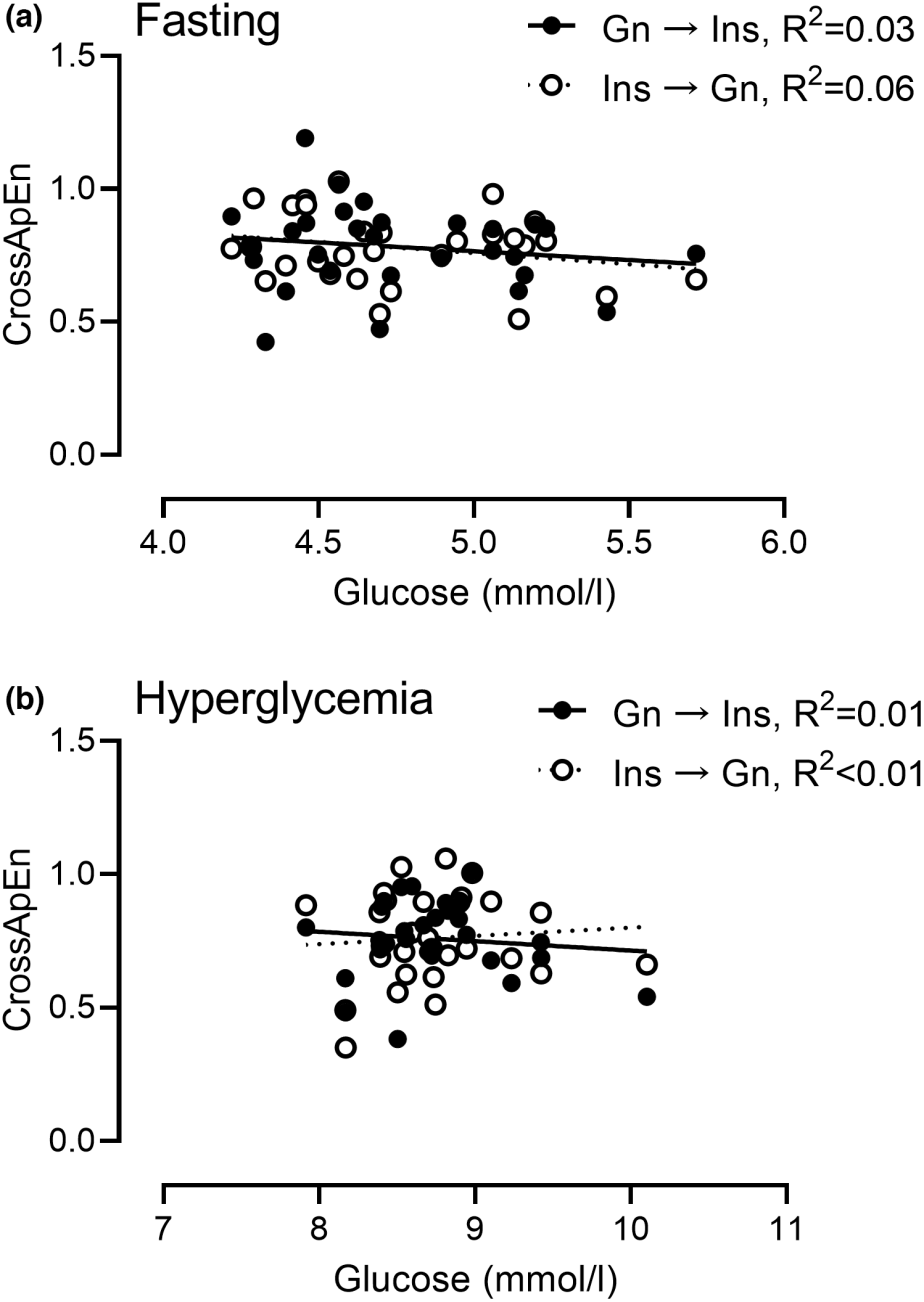
The relationship of cross‐approximate entropy during euglycemia (Panel a) and hyperglycemia (Panel b) with average blood glucose for each individual under those conditions. In both figures ● and full lines indicate the correlation between glucose and the cross‐ApEn index calculated using glucagon as a template to assess pattern reproducibility in insulin concentrations (Gn → ins), ○ and dotted lines indicate the opposite scenario (ins → Gn).

To ensure that variation in glucose concentrations did not contribute to our experimental observations, we examine the relationship of individual average glucose values during euglycemia (Panel a, *R* ^2^ ≤ 0.06) and during hyperglycemia (Panel b, *R* ^2^ ≤ 0.01). Individual variation in glucose concentrations did not affect our measurements of Cross‐ApEn between Insulin and Glucagon as shown in Figure 3.

## 4 DISCUSSION

In this study, we sought to determine the interplay of insulin and glucagon to determine if these two glucoregulatory hormones (with opposing effects on glucose metabolism) regulate one another. We report that, although there is evidence of synchronicity of insulin and glucagon, there is not enough evidence to clearly state that insulin restrains α‐cells function or vice‐versa. The concept of paracrine restraint of α‐cell secretion by insulin (Unger & Orci, 2010) (and other β‐cell factors [Rorsman et al., 1989]) is supported by experiments where isolated α‐cells respond inappropriately to hyperglycemia by secreting glucagon (Ishihara et al., 2003). Similar observations were made in islets isolated from streptozotocin‐treated animals (Ostenson, 1979). On the other hand, Pipeleers et al. showed that the insulin secretory response of reaggregated β‐cells was greater than that observed in single cells and was potentiated by endogenous (and exogenous) glucagon (Pipeleers et al., 1985).

Interest in the contribution of insulin pulse characteristics to insulin signaling (reviewed in [Laurenti, Dalla Man, et al., 2021]) and in the response to various insulin secretagogues (Porksen, Munn, Steers, Schmitz, et al., 1996; Porksen, Munn, Steers, Veldhuis, et al., 1996; Prigeon et al., 2003), led to examination of the relationship of between insulin and glucagon secretion using these methods.

Our results confirm that most subjects show an anti‐phasic relationship between glucagon and insulin during hyperglycemia, as previously observed in pigs (Meier et al., 2006) and in humans (Menge et al., 2011) after a meal. However, we also report that this relationship was inverted during fasting in most (but not all) subjects, where glucagon and insulin showed an in‐phase relationship. Values of Cross‐ApEn were independent of which hormone was used as a template for the other (follower) hormone. This suggests that the interaction between the two hormones is not driven by one another. Rather it supports the hypothesis that an external control mechanism, such as glucose concentrations or paracrine intra‐islet signaling, is more likely the driver that makes the hormones rise (or fall) in tandem. Of note, inter‐individual variation in glucose concentrations during both euglycemia and hyperglycemia does not explain differences in measures of Cross‐ApEn between the two hormones (Figure 3). Future experiments, where glucose is frequently sampled together with Insulin and Glucagon, will be necessary to determine if glucose oscillations contribute to the coordinated oscillatory secretion of α and β‐cells.

Our observation differs from previous reports where statistically lower values of Cross‐ApEn, observed in the scenario where glucagon controls insulin, implied that α‐cells regulate β‐cell secretion (Meier et al., 2006; Menge et al., 2011). It is uncertain as to whether differences in immunoassay specificity for glucagon versus other proglucagon fragments (Wewer Albrechtsen et al., 2016), the differences in sampling protocol and the effect of hormone clearance in the periphery might explain these discrepancies. Of note, at the time of analysis, the assay used was unaffected by the manufacturer's change of glucagon‐detecting antibody which significantly impairs its performance (Wu et al., 2022). The insulin and glucagon response to an oral challenge is subject to other influences such as gastric emptying and incretin hormone release (Vella & Camilleri, 2017). However, Gobl et al. recently reported that glucagon secretion after different nutrient challenges is independent of integrated insulin concentrations (Gobl et al., 2022) in keeping with data from our group (Sharma et al., 2018) and that of others (Faerch et al., 2016).

The study has several strengths, chief of which is the relatively large sample size for an invasive hepatic vein experiment in humans, hepatic vein sampling every 2 minutes, and the homogenous population studied. On the other hand, there are some limitations which need to be acknowledged. The subjects were originally recruited based on their genotype at rs7903146 in the TCF7L2 locus where the T (the type 2 diabetes‐associated) allele increased disorderliness of insulin secretion and impaired postprandial suppression of glucagon (Laurenti et al., 2020). However, genotype had no effect on the relationship of insulin with glucagon concentrations in the current analysis (data not shown). All of the subjects studied had normal fasting glucose, but a subset fulfilled criteria for impaired glucose tolerance. Glucose tolerance status did not alter the observed relationship of glucagon and insulin pulses, but we were unable to examine the effect of fasting glucose status on these parameters.

Our results are in keeping with observations that suggest the relative independence of α‐ and β‐cell function in humans (Faerch et al., 2016)—although both are regulated by similar macronutrients for example, carbohydrates and by paracrine hormones for example, somatostatin (Hauge‐Evans et al., 2009). Abnormalities of α‐cell function can be apparent in prediabetes prior to the development of overt β‐cell dysfunction and relative insulin deficiency. We conclude that insulin and glucagon function in tandem with one other but neither one controls the other. It is likely that other factors contribute to the coordinated secretion and suppression for both hormones. This has important implications for preventative and therapeutic strategies in prediabetes which, to date, have focused on β‐cell dysfunction. A better understanding of the regulation of islet function in health and disease may allow earlier intervention in prediabetes.

## AUTHOR CONTRIBUTIONS

Marcello C. Laurenti developed the method, analyzed the data and reviewed/edited manuscript. Praveer Arora contributed to the discussion and writing of the manuscript. Chiara Dalla Man developed the method and reviewed/edited manuscript. James C. Andrews researched data and ran the studies. Robert A. Rizza, Aleksey Matveyenko contributed to the discussion and reviewed/edited manuscript. Kent R. Bailey supervised the statistical analysis. Claudio Cobelli developed the method and reviewed/edited manuscript. Adrian Vella designed the study, oversaw its conduct, researched data, and wrote the manuscript. Adrian Vella is the guarantor of this work and, as such, had full access to all the data in the study and takes responsibility for the integrity of the data and the accuracy of the data analysis.

## FUNDING INFORMATION

This study was supported by US National Institutes of Health (DK78646, DK116231, DK126206), MIUR (Italian Minister for Education) under the initiative “Departments of Excellence” (Law 232/2016) and Mayo Clinic General Clinical Research Center (UL1 TR000135).

## CONFLICT OF INTEREST

The authors have declared that no conflict of interest exists.

## Data Availability

Deidentified datasets will be made available on request from the corresponding author after all necessary approvals and agreements are obtained.
